# LncRNA EIF3J-AS1 enhanced esophageal cancer invasion via regulating AKT1 expression through sponging miR-373-3p

**DOI:** 10.1038/s41598-020-70886-2

**Published:** 2020-08-18

**Authors:** Wei-tian Wei, Liang Wang, Jin-xiao Liang, Jiang-feng Wang, Qing Li, Jian Zeng

**Affiliations:** Department of Thoracic Surgery, Institute of Cancer Research and Basic Medical Sciences of Chinese Academy of Sciences, Cancer Hospital of University of Chinese Academy of Sciences, Zhejiang Cancer Hospital, No. 1 Banshan East Road, Gongshu District, Hangzhou, 310022 China

**Keywords:** Cancer, Cell biology, Genetics, Microbiology

## Abstract

Esophageal cancer (ECa) remains a major cause of mortality across the globe. The expression of EIF3J-AS1 is altered in a plethora of tumors, but its role in ECa development and progression are undefined. Here, we show that EIF3J-AS1 is up-regulated in ECa and that its expression correlates with advanced TNM stage (P = 0.014), invasion depth (P = 0.001), positive lymph node metastasis (P < 0.001) and poor survival (OS: P = 0.0059; DFS: P = 0.0037) in ECa. Functional experiments showed that knockdown EIF3J-AS1 inhibited ECa growth and metastasis through in vitro and in vivo experiments. Regarding the mechanism, EIF3J-AS1/miR-373-3p/AKT1 established the ceRNA network involved in the modulation of cell progression of ECa cells. Overall, EIF3J-AS1 may exhibit an oncogenic function in ECa via acting as a sponge for miR-373-3p to up-regulate AKT1 mRNA level, and may serve as a potential therapeutic target and a prognostic biomarker for ECa patients.

## Introduction

Esophageal cancer (ECa) is of particularly high incidence in Chinese males compared to other regions and remains a global threat to human health. Up to ~ 450,000 new cases of ECa are diagnosed annually, of which up to ~ 400,000 of those afflicted do not survive^1^. Diagnostic and therapeutic advances have been made to improve ECa therapeutics, including biopsies, chromo endoscopy and narrow-band imaging^2^. However, despite these improvements, the efficiency of ECa therapies and subsequent patient prognosis remains poor. ECa therefore remains a global health burden for which new diagnostics and effective therapeutic strategies are urgently required^3^. A further understanding of the molecular regulators of ECa tumorigenesis is required to expedite the discovery of novel anti-ECa strategies.

While lncRNAs (long noncoding RNAs; more than 200 bases long^4–8^ are not capable of translating proteins, their dysregulation is observed several cancers, such as ECa^9–12^. For instance, the process of EMT (epithelial-mesenchymal transition) is enhanced by lncRNA PVT1 via E-cadherin suppression in ECa^13^. Further, significantly enhanced level of CASC11 was observed in ECa tissues while its silencing impeded the progression of ECa by regulating KLF6 expression^14^. Previous study reported that up-regulated a novelty EIF3J-AS1 was associated with poor survival and accelerated HCC progression via targeting miR-122e5p/CTNND2 axis under hypoxia^15^. Meanwhile, EIF3J-AS1 induced by H3K27 promoted colorectal cancer proliferation and inhibited apoptosis via miR-3163/YAP1 axis^16^. Nevertheless, the role and inherent mechanisms of EIF3J-AS1 in human ECa development remains to be assessed.

In this research, we examined the increased expression of EIF3J-AS1 in ECa cell lines and tissues, the effect of EIF3J-AS1 knocked down on inhibiting the metastasis and growth of ECa cell lines in vitro and vivo. We also examined the role of EIF3J-AS1 in ECa cells’ aggressive phenotypes by miR-373-3p binding and assessing the AKT1 expression.

## Materials and methods

### Tissue specimens

Collection of a total of 182 ECa cases and equivalent adjacent tissues from esophagus were sampled from the Zhejiang Cancer Hospital. Before the experiments commenced, patients gave informed consent in writing. Immediate freezing of tissues was done in liquid N2 and kept at − 80 °C. None of the patients had undergone any treatments prior to the surgical procedure. Approval of the study was given by the Ethical Committee, of the Zhejiang Cancer Hospital. All experiments were performed in accordance with the relevant guidelines and regulations.

### Cell culture

Human endothelial HET-1A cells and ECa cells including EC9706 and TE-1 were grown in DMEM + 10% FBS at 37 °C. ECa ECA109 and TE-8 cells were grown in RPMI-1640 + 10% FBS at 37 °C. For All mentioned transfection reagents were obtained by GenePharma Co., Ltd (Shanghai, China).

### Real-time PCR

RNA was isolated from cell lines or paraffin-embedded tissue using a miRNeasy FFPE kits (Qiagen) or miRNeasy Mini kits (Qiagen). cDNA was generated using a miScript II RT kit (Qiagen) from 1 µg of input RNA or miRNA. cDNA synthesis was as follows: 37 °C for 60 min, 95 °C for 5 min. The miScript SYBR Green PCR kit was used for qRT-PCR. Reactions were performed as follows: 95 °C for 15 min; 40 cycles of 94 °C for 15 s, 55 °C for 30 s, and 70 °C for 30 s on a 7,500 Fast Real-Time PCR platform. Values were normalized to GAPDH and U6 and quantified. All reactions were performed in triplicate. The primers were listed below:

- EI3J-AS1: forward 5′-GCCACAATGATACAGGTT-3′
- reverse 5′-GCCAGTGACCTGTCCACCC-3′;
- GAPDH: forward 5′-GGGAAACTGTGGCGTGAT-3′
- reverse 5′-GAGTGGGTGTCGCTGTTGA-3′;
- U6: forward 5′-CTCGCTTCGGCAGCACATATACT-3′
- reverse 5′-ACGCTTCACGAATTTGCGTGTC-3′
- MiR-373-3p: forward 5′-ACACTCGCTGCCTGAATTG-3′
- reverse 5′-GTGCAGGGACCGAGGT-3′
- AKT1: forward 5′-ACCTCTAACCTACCTCA-3′
- reverse TCCAGAAGAGGTACTA-3′.

### Cell proliferation assays

CCK-8 assays were used to assess ECa cell proliferation. Cells were co-transfected as described in 96-well plates (1,000 cells/well) for 24 h, and 10 μl of CCK-8 was added to the wells in normal culture conditions for 3 h. Absorbances at 450 nm were read on a Bio-Rad microplate reader.

### Transwell assay

For migration/invasion assays, 12-well plates containing Transwell inserts (Corning, MA, USA) with 8 μm pore sizes were used and performed as previous^17^.

### LncRNA Localization

The LncRNA Localization assays were performed as previous^18^. To localize lncRNA, Cytoplasmic and Nuclear RNA Purification Kit (Amyjet) helped to isolate and extract the nuclear and cytoplasmic RNA of ECa cells, and qRT-PCR helped to measure the expression of EIF3J-AS1 in above two types of RNA. GAPDH was the cytoplasm control and U1 was the nuclear control.

### RNA immunoprecipitation (RIP) assay

A Magna RIP RNA-Binding Protein Immunoprecipitation Kit (Millipore, Bedford, MA, USA) was used to perform the RIP assay to evaluate the interaction between EIF3J-AS1 and miR-373-3p in ECa cells and performed as previous^19^.

### Reporter assay for luciferase

Culturing of ECa cell was done in plates of 24 wells. After incubating for one full day, transfection of the reporter vector pmirGLO from Promega harboring the WT (wild-type) or MUT (mutant) EIF3J-AS1 was done into ECa cell combined with miR-373-3p. The miR-373-3p and AKT1 relationship was explored by culturing ECa cell in plates of 24 wells. After incubation for one full day, the transfection of the reporter vector pmirGLO harboring ZEB1 (WT or MUT) was done into ECa cell combined with miR-373-3p. Post-transfection of two full days, estimation of luciferase activity was done through Reporter System of Dual-luciferase from Promega.

### In vivo metastasis assays

In vivo assessments were performed as previous^20^ in male nude mice aged 6 weeks (Beijing Vitonlihua Experimental Animal Technology Co. Ltd, Beijing, China). Animals were housed in specified cages that were approved by the national animal guidelines of our institute. All animal experiments were approved by ethics committee of Zhejiang Cancer Hospital. 1 × 10^6^ indicated Eca cells were subcutaneously injected into the flanks of 5 mice from (n = 5 for each group). The tumor volumes and weights were measured at the indicated time points (7, 14, 21, and 35 days). Five weeks following injection, mice were humanely killed by CO2 in accordance with ethical study requirements. For in vivo metastatic growth in the lungs, Mice were injected with indicated treated Eca cells (4 × 10^5^ cells, 5 mice per group) in the tail vein to produce the pulmonary metastasis model. Ten weeks following injection, mice were humanely killed by CO2 in accordance with ethical study requirements and H&E stained to identify the presence of metastatic foci in the lungs. None anaesthetics were used during animal experiments.

### Construction of siRNA, plasmids, miRNA mimic, inhibitor, and transient transfection

Three shRNA sequence for knocking down EIF3J-AS1(shRNA#1: 5′-GGAACUCCCUGCCUUCAUCCUUU-3′; shRNA#2: 5′-AGGCUGGAAACUGCCACCAACUUAA-3′; shRNA#3: 5′-GGCCCGUUUUGGGAACUAACCCAA-3′) and the corresponding negative control (sh-NC: 5′-UUUCUCCGAACGUGUCACGUTT-3′) were synthesized by GenePharma (Shanghai, China). The AKT1 expressing plasmid (LV-AKT1) and the corresponding negative plasmid vector (vector) were provided by GenePharma (Shanghai, China). The miR-373-3p Mimic and NC Mimic; miR-373-3p Inhibitor and NC Inhibitor were synthesized by GenePharma (Shanghai, China). Lipofectamine 3,000 kit (Invitrogen) was used in transient transfection according to the manufacturer’s instructions and performed as previous^21^.

### Statistical analysis

Data are shown and the mean ± SD analyzed via SPSS 17.0. Inter-group differences were assessed via a student’s t-test. Multiple groups were compared using a one-way ANOVA analysis of variance. Kaplan Meier (KM) curves were plotted for survival analysis, and groups were compared via log-rank assessments (n = 3 for all). P-values < 0.05 were deemed significant differences.

## Results

### EIF3J-AS1 is increased in ECa

**Figure 1:**
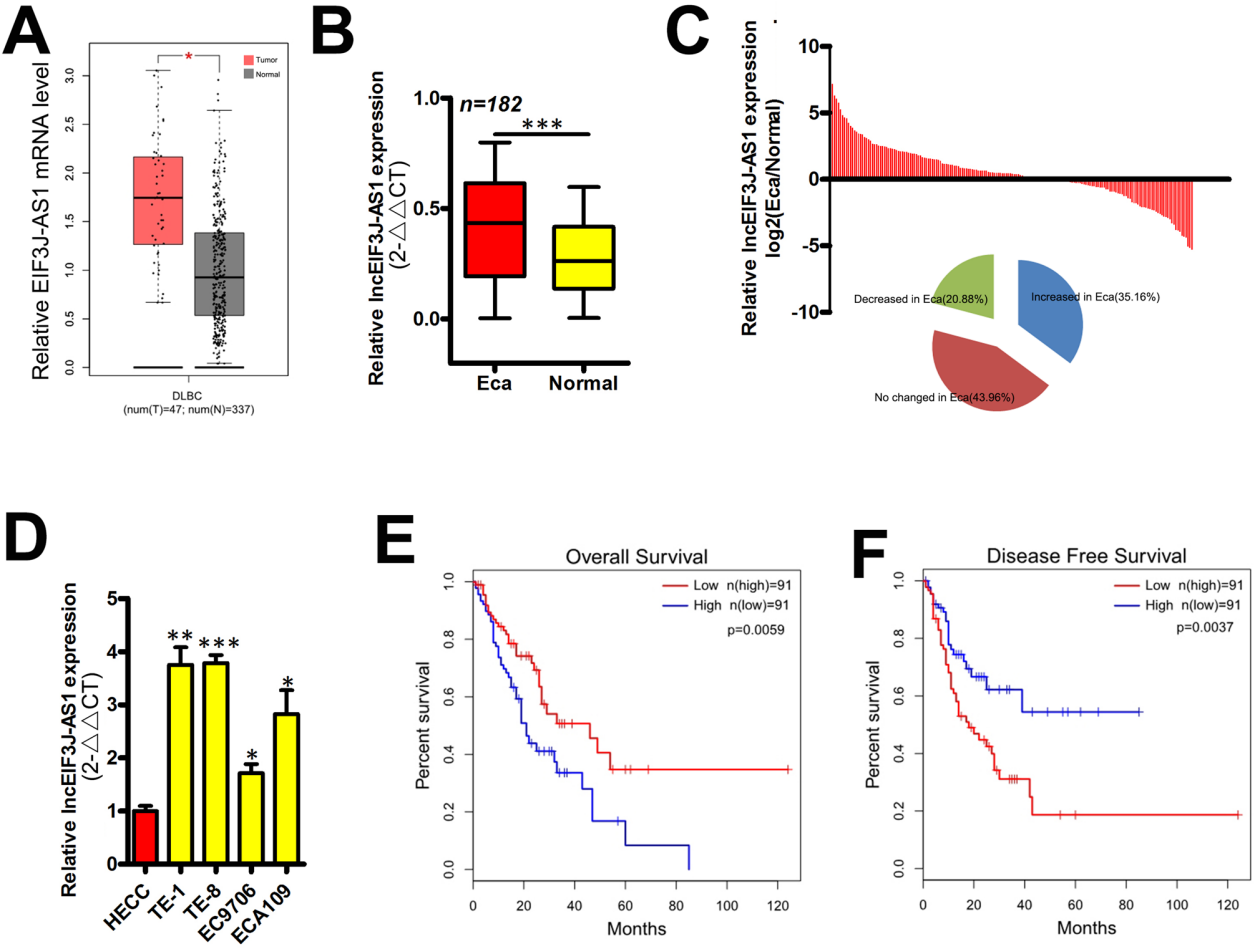
EIF3J-AS1 levels are increased in ECa. (**A**) Expression of EIF3J-AS1 in ECa and paired normal tissue samples from the GEPIA data. (**B**) Expression of EIF3J-AS1 was measured in ECa and paired normal tissue samples via qRT-PCR. (**C**) Histogram and pie chart of the proportions of Eca samples in which EIF3J-AS1 expression was up-regulated (64/182, 35.16%, blue), down-regulated (38/182, 20.88%, green), or no change (80/182, 43.96%, red). Log2 (T/N) expression value > 1 as higher expression, which <  − 1 as lower expression, and between − 1 and 1 as no significant change. (**D**) Expression of EIF3J-AS1 was measured in ECa and control cell lines. (**E**, **F**) Overall survival (OS) and Disease-free survival (DFS) in ECa patients. Kaplan Meier (KM) curves were plotted for survival analysis. p-value *< 0.05; **< 0.01; ***< 0.001.

From the GEPIA (Gene Expression Profiling Interactive Analysis) data indicated that EIF3J-AS1 as increased in ECa tissues compared to normal tissues (Fig. 1A, Fold change > 1.5; p-value < 0.05). We next investigated EIF3J-AS1 levels in ECa *vs.* non-cancer cells and tissues via qRT-PCR analysis. EIF3J-AS1 expression was found to be higher in ECa tissues compared to healthy tissues (Fig. 1B) with 64/182(35.16%) of ECa samples showing higher levels of the lncRNA (Fig. 1C). RT-PCR analysis further showed that EIF3J-AS1 was up-regulated in ECa cell lines vs. non-ECa HECC cells. Amongst the cell-types, EIF3J-AS1 showed the highest levels of expression in TE-1 and TE-8 cells (Fig. 1D). These data confirmed that EIF3J-AS1 expression is enhanced in ECa tissues and cells.

### High levels of EIF3J-AS1 expression correlate with aggressive clinicopathologic features and poor survival in ECa

To investigate the correlation of EIF3J-AS1 expression and the clinicopathological characteristics of ECa, subjects were divided into high- and low-subgroups according to the median EIF3J-AS1 expression levels measured in ECa tissues (as a cut-off value). Table 1 shows that high levels of EIF3J-AS1 positively correlated with advanced TNM staging (P = 0.014), invasion depth (P = 0.001) and lymph node metastasis (P < 0.001). To explore the interplay between EIF3J-AS1 expression and the prognosis of ECa patient’s, follow-up data was compiled. KM curves suggested that high levels of EIF3J-AS1 were associated with a poor ECa prognosis, poor OS, and low rates of DFS (Fig. 1E,F). These data confirmed that EIF3J-AS1 expression correlates with pathological stage, distant metastasis and survival in ECa patients.

**Table 1:** The correlation between clinicopathological parameters and LNCEIF3J-AS1 expression in human esophageal cancer.

Clinical features	Total (N = 182)	LNCEIF3J-AS1		*p*-value
		Low (N = 91)	High (N = 91)	
Age (years)				0.105
< 60	54 (29.7%)	22 (12.1%)	32 (17.6%)	
≥ 60	128 (70.3%)	69 (37.9%)	59 (32.4%)	
Gender				0.765
Male	102 (56%)	52 (28.6%)	50 (27.5%)	
Female	80 (44%)	39 (21.4%)	41 (22.5%)	
Differentiation grade				0.432
Well	61 (33.5%)	28 (15.4%)	33 (18.1%)	
Moderate + poor	121 (66.5%)	63 (34.6%)	58 (31.9%)	
TNM stage				**0.014**
I + II	114 (62.6%)	65 (35.7%)	49 (26.9%)	
III	68 (37.4%)	26 (14.3%)	42 (23.1%)	
Depth of invasion				**0.001**
T1 + T2	85 (46.7%)	54 (29.7%)	31 (17%)	
T3 + T4	97 (53.3%)	37 (20.3%)	60 (33%)	
Lymph node metastasis				** < 0.001**
No	120 (65.9%)	79 (43.4%)	41 (22.5%)	
Yes	62 (34.1%)	12 (6.6%)	50 (27.5%)	

Pearson chi-square test was used for comparison between subgroups.

Bold values indicate *p*-value < 0.05.

### Effects of EIF3J-AS1 Knockdown on ECa cells growth and metastasis

**Figure 2:**
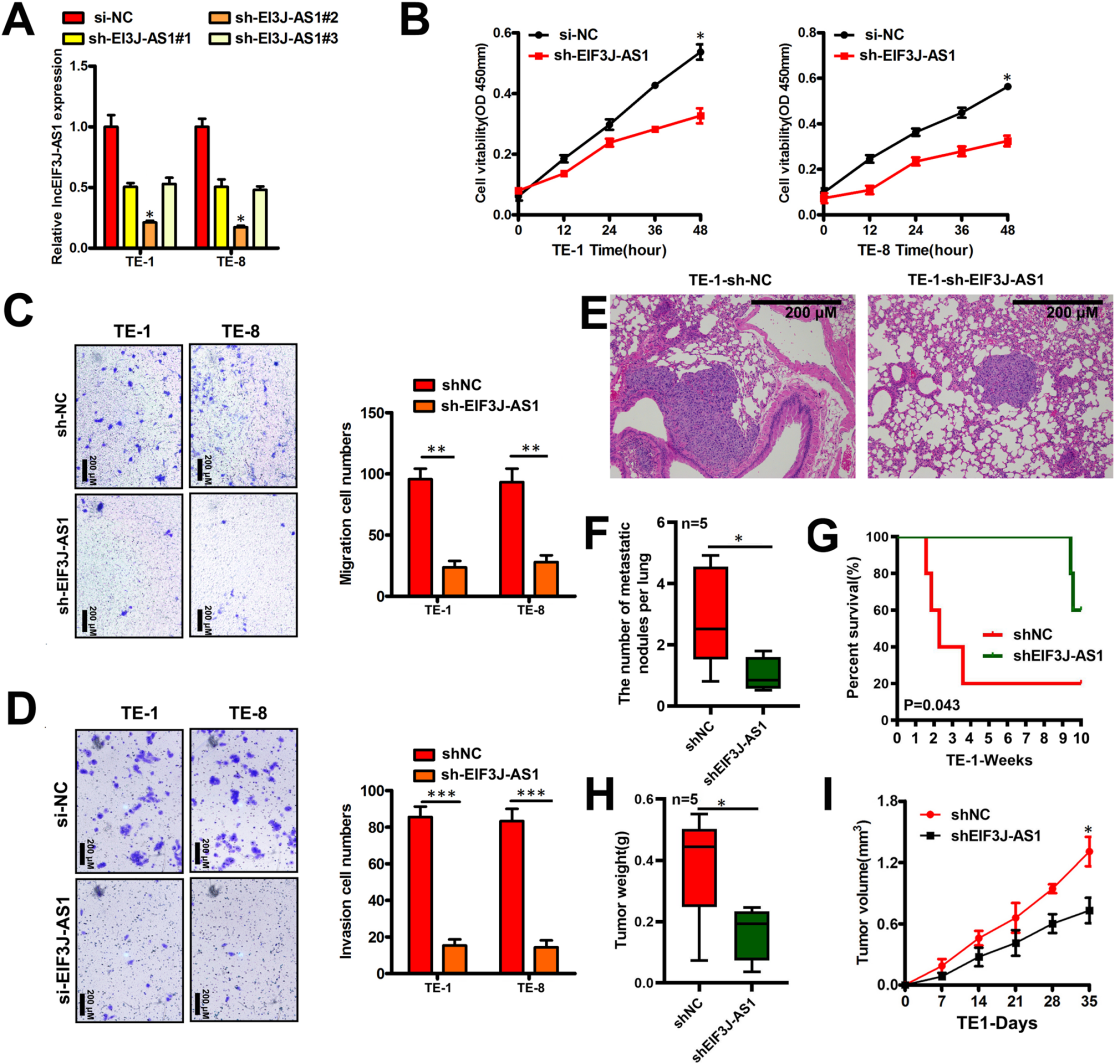
Effects of EIF3J-AS1 Knockdown on ECa cells growth and metastasis. (**A**) RT-qPCR analysis of EIF3J-AS1 expression in indicated ECa cells after the transfection of sh-EIF3J-AS1 or sh-NC. (**B**) The proliferation of EIF3J-AS1 deficient-indicated ECa cell was detected using the Cell Counting Kit-8 assay. (**C**, **D**) Transwell assays were performed to assess the migratory and invasive abilities of Indicated ECa cells following sh-EIF3J-AS1 or sh-NC transfection. (**E**) H & E staining of mouse lung tissues from indicated treated group. (**F**) Numbers of metastatic foci observed in each group (n = 5). (**G**) Comparisons of the OS curves of mice by Kaplan–Meier survival analyses. (**H**, **I**) Indicated treated ECa cells were subcutaneously injected into nude recipient mice (n = 5). *P < 0.05; **P < 0.01; ***P < 0.001.

To investigate the biology function role of EIF3J-AS1in ECa, we chose TE-1and TE-8 cells which have relative higher level of EIF3J-AS1 for further experiments. Through shRNA, sh-EIF3J-AS1#2 expression was significantly decreased in these cell lines (Fig. 2A). CCK8 assay showed EIF3J-AS1 downregulation impaired the proliferation of TE-1and TE-8 cells (Fig. 2B). Next, cell migration and invasion were assessed by Transwell assay. Results showed that EIF3J-AS1 knockdown reduced migration and invasion of TE-1and TE-8 cells (Fig. 2C,D). Following, we injected indicated treated EcaTE-1 cells (sh-NC vs. sh-EIF3J2-AS1group) into the lateral tail vein of nude mice, the results indicating that knocking EIF3J2-AS1 group showed fewer numbers of metastatic foci and higher survival rate compared to sh-NC group (Fig. 2E–G). Meanwhile, tumor formation was delayed in sh-EIF3J2-AS1group knockdown group compared to sh-NC group (Fig. 2H,I). Taken together, above results indicate that EIF3J-AS1 was an oncogene in ECa.

### MiR-373-3p was regulated by EIF3J-AS1 in ECa cells

**Figure 3:**
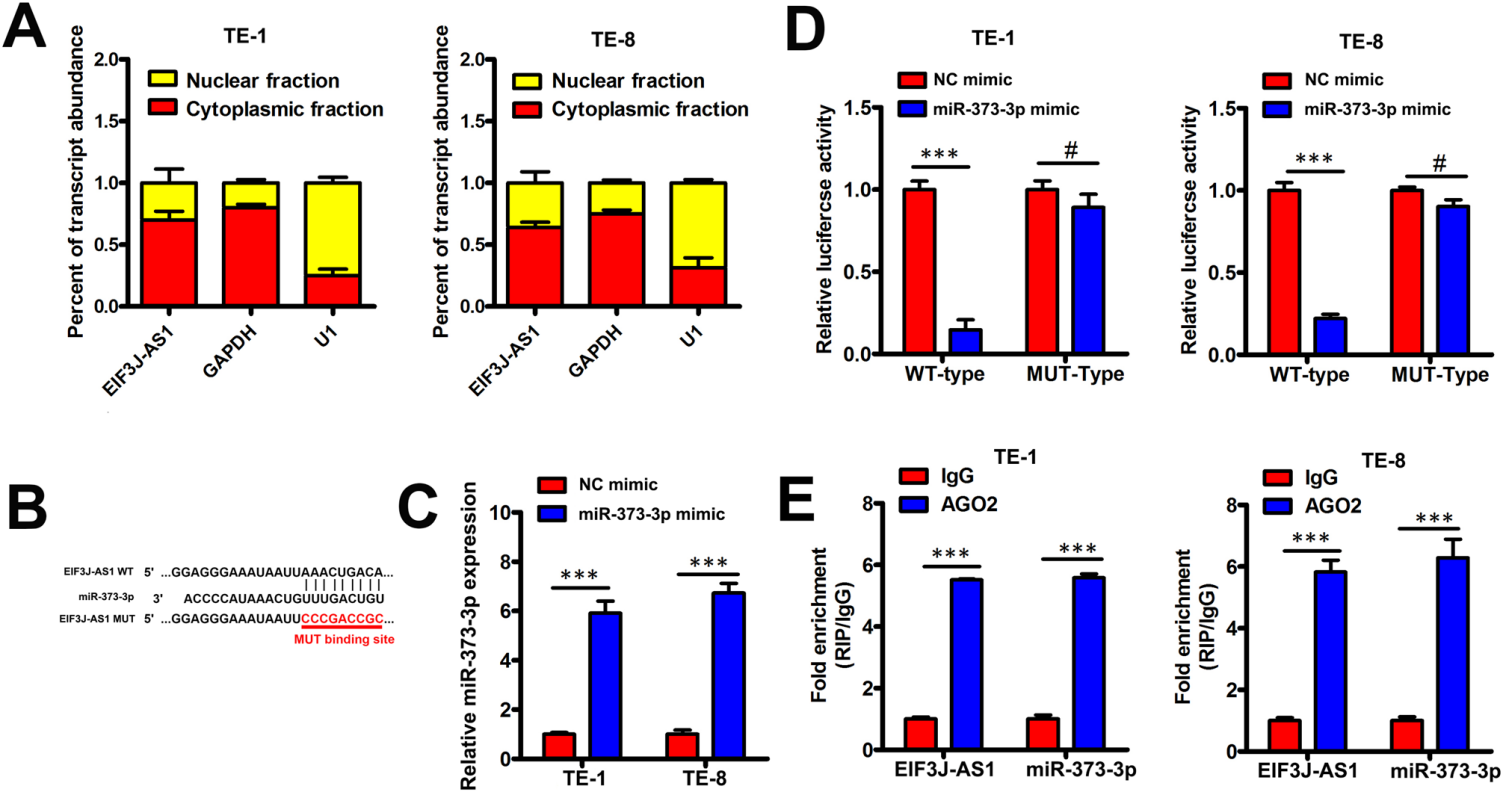
MiR-373-3p was regulated by EIF3J-AS1 in ECa cells. (**A**) As shown in nuclear RNA fractionation and cytoplasmic experiments, EIF3J-AS1 could be mainly found in cytoplasm of TE-1 and TE-8 cells. (**B**) Bioinformatic analyses predicted an interaction between miRNA-373-3p and lncEIF3J-AS1. (**C**) The expression of lncEIF3J-AS1 in TE-1 and TE-8 cells was assessed following mir-373-3p mimic or NC mimic transfection. (**D**) Wild type (WT)-lncEIF3J-AS1 or mutant (MUT)-lncEIF3J-AS1 were co-transfected into Eca cells together with mir-373-3p mimic or NC mimic. After 48 h, luciferase activity was assessed. (**E**) Increased EIF3J-AS1 and miR-373-3p levels were evident in Ago2-containing immunoprecipitates relative to precipitates prepared using a control IgG. p-value *< 0.05; **< 0.01; ***< 0.001.

Acting as competing endogenous RNAs (ceRNAs), lncRNAs may binding to specific miRNAs to suppress target mRNA genes expression^20^. To verify EIF3J-AS1 acted as ceRNA in Eca, we performed Nuclear/cytoplasmic fraction assay and Fig. 3A results showed that EIF3J-AS1 localized in indicated Eca cell lines cytoplasm, indicating that EIF3J-AS1 could act as a ceRNA. To investigate this, the starBase tool (https://starbase.sysu.edu.cn/) was applied to screen the potential miRNAs that interact with B4GALT1-AS1 and the potential target miRNA of EIF3J-AS1 was miR-373-3p (Fig. 3B). Firstly, we increased miR-373-3p level in indicated Eca cell lines (Fig. 3C). Next, co-transfection of pmirGLO reporter vector with either EIF3J-AS1-WT (WT EIF3J-AS1-AS1) or EIF3J-AS1-MUT (MUT) combined with miR-373-3p mimic was done in ECa cell. We next increased miR-373-3p level by transferring miR-373-3p mimic in ECa cells. As per Fig. 3D, the activity of luciferase reduced remarkably due to miR-373-3p in indicated Eca cells transfected with EIF3J-AS1-WT, although it had no inhibitory effect on the activity of luciferase in EIF3J-AS1-MUT transfected cells. Next, the miR-373-3p and EIF3J-AS1 relationship was confirmed through the RIP assay. The miR-373-3p and EIF3J-AS1 combination complex precipitated in AGO2 (Fig. 3E). Therefore, EIF3J-AS1 negatively regulates by miR-373-3p by binding with miR-373-3p in Eca cell lines.

### EIF3J-AS1-mediated ECa metastasis via miR-373-3p/AKT1 axis

**Figure 4:**
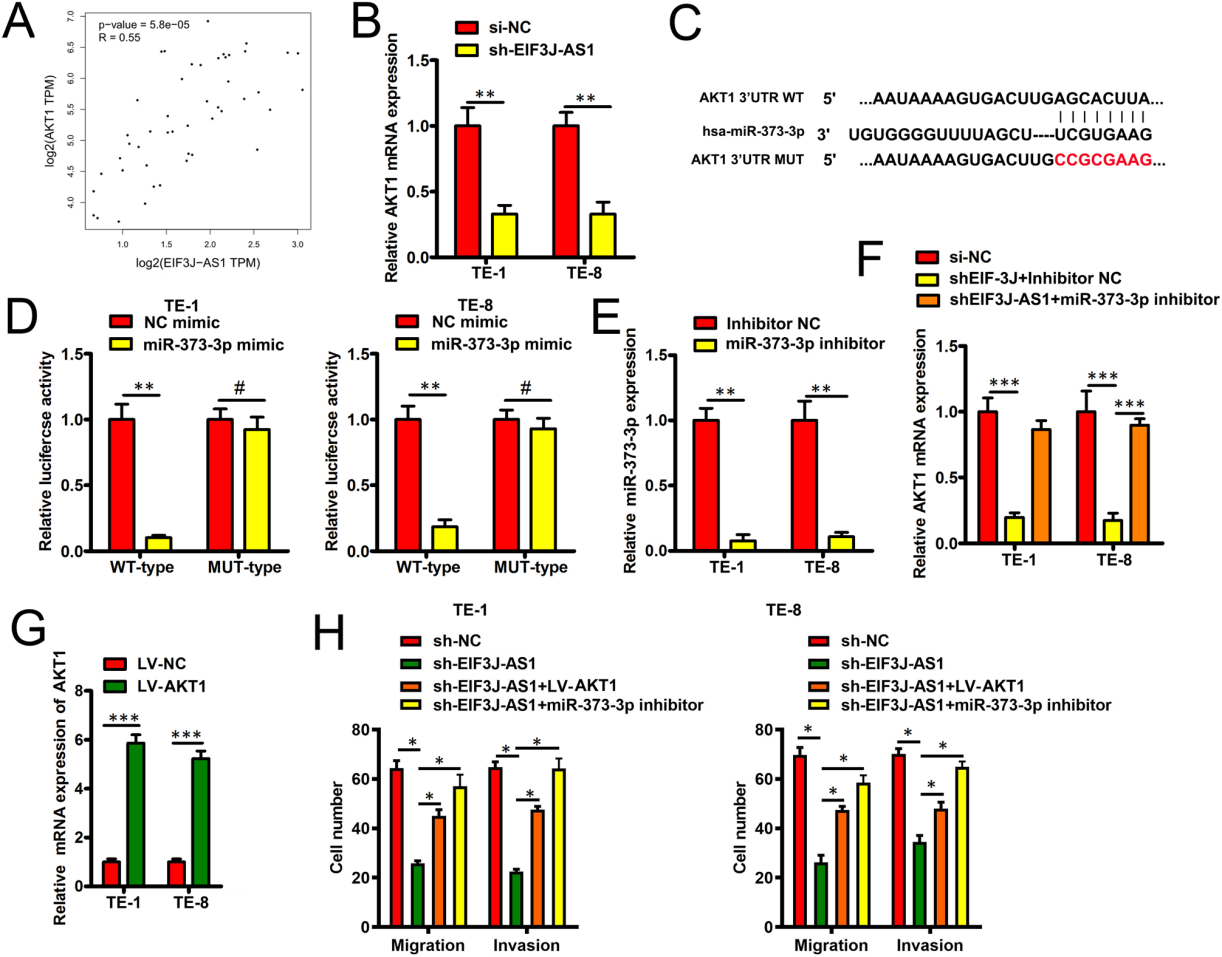
EIF3J-AS1-Mediated ECa metastasis via miR-373-3p/AKT1 axis. (**A**) GEPIA tool indicted that a strong postively correlation between the expression of EIF3J-AS1 and AKT1 in these ECa tissue samples. (**B**) AKT1 mRNA level expression analysis in indicated E Ca cells with EIF3J-AS1 knock down. (**C**) Bioinformatic analyses predicted an interaction between miRNA-373-3p and AKT1. (**D**) miR-373-3p mimic transfection reduced the activity of a WT AKT1 3′-UTR luciferase reporters in ECa cells. In contrast, no changes were observed upon EIF3J-AS1 mimic transfection in cells co-transfected with a reporter in which these 3′-UTR binding sites had been mutated. (**E**, **F**) miR-373-3p and AKT1 mRNA was performed by RT-qPCR in indicated ECa cells. (**G**) AKT1 mRNA was performed by RT-qPCR in indicated ECa cells. (**H**) Transwell assays were performed to assess the migratory and invasive abilities of Indicated ECa cells. *P < 0.05; **P < 0.01; ***P < 0.001.

To study the regulation of ECa via EIF3J-AS1, we found that EIF3J-AS1 was positively co-expression with AKT1 from the GEPIA (Gene Expression Profiling Interactive Analysis) data (Fig. 4A). Meanwhile, in ECa cells knock down EIF3J-AS1, a marked decrease in AKT1 mRNA level was observed (Fig. 4B). Furthermore, targetscan3.0 identified miR-373-3p as a putative binding target for AKT1 based on sequence complementarity (Fig. 4C), indicating that EIF3J-AS1 may act as ceRNA to sponge miR-373-3p to enhance to AKT1 inducing ECa progression. We then used a luciferase reporter assay approach to determine whether miR-373-3p was able to bind to its predicted target sequence in the AKT1 3′-untranslated region (UTR). We found that miR-373-3p mimic transfection was able to inhibit the activity of a wild-type (WT) AKT1 3′-UTR luciferase reporter, whereas it had no effect on a reporter in which its putative binding site had been mutated, consistent with the ability of miR-373-3p to directly bind to this 3′-UTR region (Fig. 4D). To explore whether EIF3J-AS1 regulates AKT1 expression through sponging miR-373-3p, sh-EIF3J plus miR-373-3p inhibitor or inhibitor NC was introduced into ECa cells. First, the efficiency of miR-373-3p inhibitor transfection was verified using RT-qPCR. The data revealed that transfection with miR-373-3p inhibitor resulted in a significant decrease in miR-373-3p expression in ECa cells (Fig. 4E). Furthermore, the downregulation of AKT1 mRNA (Fig. 4F) caused by EIF3J-AS1 knock down was reversed in ECa cells through miR-373-3p inhibitor re-introduction. To explore whether EIF3J-AS1 regulates ECa cells migration and invasion through AKT1, sh-NC, sh-EIF3J-AS1 or EIF3J-AS1 + LV-AKT1 was introduced into ECa cells. First, the efficiency of LV-AKT1 transfection was verified using RT-qPCR. The data revealed that transfection with LV-AKT1 resulted in a significant increase in AKT1 expression in ECa cells (Fig. 4G). Furthermore, the decreased migration and invasion ability of ECa cells caused by EIF3J-AS1 knock down was reversed in ECa cells through up-regulated AKT1or decreased AKT1 via miR-373-3p inhibitor (Fig. 4H). Taken together, EIF3J-AS1-Mediated ECa progression by via miR-373-3p/AKT1 axis.

## Discussion

The dysregulation of lncRNAs is increasing linked to tumorigenesis, with both oncogenic and tumor suppressor functions reported^22–25^. In this study, higher levels of EIF3J-AS1 expression were observed in ECa tumors and cells, which was associated with advantaged TNM staging (P = 0.014), invasion depth (P = 0.001), metastasis (P < 0.001), and poor survival. Moreover, EIF3J-AS1 enhanced the proliferation and metastatic phenotypes of ECa cells through its ability to sponge miR-373-3p to increase AKT1 level. These findings collectively highlight EIF3J-AS1 is a key regulator of ECa tumorigenesis.

LncRNAs act as quenchers of miRNA to regulate tumor progress. Currently, LncRNAs act as quenchers of miRNA to regulate tumor progress^26^. For example, the proliferative, invasiveness, and migratory capacity of pancreatic cancer cell lines are regulated by LncRNA linc00673 by sequestering miR-504^27^. Previous study indicated that EIF3J-AS1 may induce HCC progression via sponging miR-122e-5p^15^. Following, we wonder whether EIF3J-AS1 exerts roles through sponging other miRNAs to regulate AKT1 level. In our findings revealed a ceRNA model including EIF3J-AS1, miR-373-3p, and AKT1 in ECa cells. MiR-373-3p, transcribed from chromosome 19q13.42, with the function of cancer inhibition or promotion, belongs to the miRNAs-371-372-373 family and is commonly deregulated in many cancers^28^. And recently, it is proven that miR-373-3p plays a vital role in the regulation of breast cancer^29,30^, testicular germ cell tumors^31^, etc. Nevertheless, the role and inherent mechanisms of miR-373-3p in human Eca development remains to be assessed. Here, we could confirm that the one of targets of miR-373-3p is AKT1. We could prove the suppression of AKT1 by miR-373-3p in ECa cell lines. AKT1 was an oncogene in several cancer diseases including osteosarcoma, pancreatic ductal adenocarcinoma, colorectal cancer, and breast cancer^32–35^.Recently, lncRNAs have been implicated in the miRNA/AKT1 axis modulation in human cancers. For instance, the LINC00324 counteract miR-615-5p and enhance the expression of AKT1 in lung adenocarcinoma^36^. The lncRNA RNCR3 participates in the malignant transformation of colorectal cancer through miR-1301-3p/AKT1 axis interaction^37^. The current data revealed that AKT1 expression was enhanced by EIF3J-AS1 in ECa cell lines by sequestering endogenous miR-373-3p. The rescue experiments further showed the modulation of malignancy, growth, and metastatic related traits of ECa cells by EIF3J-AS1 through the miR-373-3p/AKT1 axis. Our study indicated a novelty EIF3J-AS1/miR-373-3p/AKT1 axis in ECa progression.

To summarize, our study proves the overexpression of lncRNA EIF3J-AS1 in ECa of humans. The ECa cell lines advancement and tumorigenesis are promoted by EIF3J-AS1 by miR-373-3p sponging and positively regulating AKT1 expression. This study shows a fresh perspective on the molecular basis for comprehending ECa progression in terms of the oncogenic characteristic of EIF3J-AS1.
